# Quantitation of Apremilast in Beagle Dogs Plasma by UPLC-MS-MS and Its Application to Pharmacokinetic Studies

**DOI:** 10.1155/2021/8881076

**Published:** 2021-01-15

**Authors:** Wei Xiong, Ling Wang, Haiyan Zhang, Xiaoqiu Tao, Xuehua Jiang, Zejuan Liu, Jiajia Zhao, Wenwen Xu

**Affiliations:** ^1^Department of Clinical Pharmacy, Pharmacy School of Sichuan University, Chengdu, Sichuan 610041, China; ^2^Sichuan Tobacco Quality Supervision and Testing Station, Chengdu, China

## Abstract

A sensitive and selective ultra-performance liquid chromatography-tandem mass spectrometry (UPLC-MS-MS) method for the determination of apremilast in beagle dog plasma has been developed and successfully validated in the current study. Clopidogrel was employed as an internal standard (IS), and liquid-liquid extraction by tert-butylmethyl ether was used for sample preparation. Chromatographic separation was achieved on a UPLC BEH Shield RP18 column (50 mm × 2.1 mm, 1.7 *μ*m) with 5 mM ammonium formate water and 5 mM ammonium formate methanol as the mobile phase with gradient elution. Calibration plots were linear in the range of 2–3000 ng/mL for apremilast in beagle dog plasma. Mean recoveries of apremilast in beagle dogs plasma ranged from 87.4% to 97.4%. The intrarun and interrun precision was less than 6% and 9%, respectively, with the accuracy between 92.4% and 101.1%. The method has also been successfully applied in the pharmacokinetics study of apremilast. The mean t1/2Z was 5.41 h for 30 mg·day^−1^ for beagle dogs after oral administration. The AUC0-t increased linearly from 3.51 to 1802.13 *μ*g L^−1^*∗*h after administration of single doses.

## 1. Introduction

Psoriasis and/or psoriatic arthritis (PsA), prevalent in an estimated 2-3% of worldwide population [1, 2], is a kind of the chronic inflammatory disease process driven by overproduction of inflammatory mediators released by innate and adaptive immune cells [3, 4]. Phosphodiesterase 4 (PDE4) is a principle enzyme dominant in immune cells and modulates the production of these cytokinins such as interleukin- (IL-) 17, IL-23, and tumour necrosis factor (TNF), as well as anti-inflammatory mediators such as IL-10. Apremilast is an oral PDE4 inhibitor recently approved by FDA as the first-line treatment of PsA in the USA and by the Drug Controller General of India for marketing in India [5].

The usual presentation of apremilast is apremilast tablets, marketed in various doses such as10 mg, 20 mg, and 30 mg [5]. Apremilast is extensively metabolized by two kind of metabolic pathways: CYP-mediated oxidative metabolism (mainly by CYP3A4 enzyme) and subsequent glucuronidation or non-CYP-mediated hydrolysis. Only 3% and 7% of the administered dose is excreted unchanged in urine and feces, respectively [6, 7]. Moreover, as a substrate of CYP3A4 enzyme, apremilast may interact with CYP3A4 enzyme inducers, and a reliable validated assay is necessary for the therapeutic monitoring and to avoid any possible pharmacokinetic (PK) interactions.

Several methods were developed for the determination of apremilast and its metabolites in biological fluids samples, including the differential scanning calorimetry [8], X-ray powder diffraction method [9], Fourier transfer infrared spectroscopy [10], high performance liquid chromatography [11, 12], or liquid chromatography combined with mass spectrometry (LC-MS/MS) [6, 13, 14]. Recent determination of apremilast and its metabolites in biological fluids by ultra-performance liquid chromatography-tandem mass spectrometry (UPLC-MS-MS) was performed, and it was used to assess the accuracy of apremilast determination in serum, plasma, or blood sample of rat and pharmacokinetic of apremilast after oral administration [15, 16]. The reported C_max_ value of apremilast is 333 ng/ml after oral administration of recommended 20 mg dose [17], and it is needed to develop a sensitive method with high calibration range for the therapeutic monitoring.

Therefore, the aim of this study was to develop and validate an UPLC-MS-MS method for the determination of apremilast in plasma with high calibration range. It was applied to study the pharmacokinetic of apremilast after its oral administration at three different doses in beagle dogs.

## 2. Experimental

### 2.1. Chemicals and Animals

Apremilast (1 mg, purity 98.5%) and the internal standard clopidogrel (chemical purity 99%) were supplied by Toronto Research Chemicals (Toronto, Canada). Formic acid solution for HPLC (49–51%) and ammonium formate were obtained from Sigma-Aldrich Corporation (St. Louis, USA). HPLC grade acetonitrile and methanol were obtained from Fisher Scientific (New Jersey, USA). Beagle dogs were provided by the Animal Center at the West China School of Pharmacy, Sichuan University.

### 2.2. Preparation of Standard and Quality Control Samples

The apremilast and clopidogrel (internal standard, IS) standard solutions were prepared by solubilizing the proper amount in methanol. Fresh solutions were prepared weekly. The concentration range of calibration ranged from 2 ng·mL^−1^ to 3000 ng·mL^−1^. QC samples were prepared in a similar manner at three concentrations. All solutions and QC samples were prepared in amber flasks and stored at −20°C before use.

### 2.3. Sample Preparation Procedure

A 200 *μ*L aliquot of plasma was mixed with 50 *μ*l of 5% formic acid, 1 mL tert-butylmethyl ether, and 10 *μ*L IS (1 *μ*g·ml^−1^). After carefully vortex-mixed for 3 min (VtexMixer VX200, Labnet International, USA), the mixture was centrifuged at 12,000 rpm for 3 min. The clarified supernatant was transferred to a new polypropylene tube and evaporated to dryness under nitrogen at 45°C (Pressured Gas Blowing Concentrator TurboVap II;, Caliper, American). The residue was reconstituted in 100 *μ*L methanol, vortex-mixed, and centrifuged again under the abovementioned conditions. A 10 *μ*L of supernatant was introduced into 1.5 mL HPLC microvials maintained at +4°C until UPLC-MS-MS analysis.

### 2.4. Equipment

The chromatograph used was an Acquity Ultra-Performance LC System consisted of a quaternary pump, a degasifier for the mobile phase and an autosampler, and a temperature controller for the column module (Waters, USA). Separations were performed on an Atlantis UPLC BEH Shield RP18 column (50 mm × 2.1 mm, 1.7 *μ*m particle size, Water, USA) placed in a temperature controller for the column module. The UPLC system was coupled to a triple quadrupole (TSQ) Xevo TQTM mass spectrometer (MS) from WATERS Inc., with an *Z* spray electrospray ionization (ESI) interface and operated with Analyst software package (Version 4.1, Waters, USA).

### 2.5. LC-MS-MS Conditions

The mobile phase used for chromatography was composed of 5 mM ammonium formate in water (solvent A) and methanol (solvent B). The gradient condition was as follows: 0∼0.50 min, 70% A; 0.50∼0.52 min, 70% A∼5% A; 0.52∼2.00 min, 5% A; 2.00∼2.10 min, 5% A∼70% A; and 2.10∼4.00 min, 70% A.

**Figure 1:**
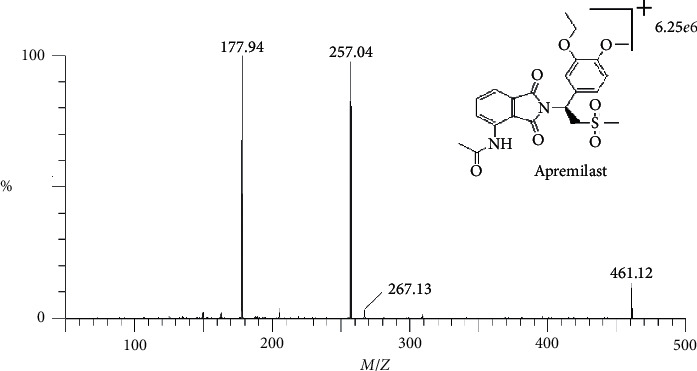
Mass spectra of product ions of apremilast and its IS.

The conditions of tandem mass were as follows: ESI ionization in the positive mode, capillary voltage: 3.00 kV, and source temperature: 350°C. The collision gas (argon) flow rate was 0.16 ml·min^−1^. Cone and desolution gas (nitrogen) flow rates were 50 and 800 Lh^−1^, respectively. MS was carried out in the multiple reactions monitoring mode (MRM). Apremilast with chemical structure is shown in Figure 1. These ion transition pairs were 461.0/178.0 (cone: 26 and CE: 26) and 461.0/257.2 (cone: 26 and CE: 9) for apremilast and 322.1/184.0 (cone: 22 and CE: 20) for clopidogrel as internal standard.

### 2.6. Method Validation Protocol

The method was validated according to the FDA Bioanalytical Method Validation Guidance [18] which includes linearity, limits of quantification (LLOQ) precision, accuracy, recovery, selectivity, the matrix effect, and stability.

The method specificity was assessed by analyzing six blank plasma samples from different sources. The “cross-talk” between MRM transitions was evaluated by analyzing the different blank samples.

In order to investigate the linearity, a series of calibration standards were prepared by analyzing known concentrations of apremilast and the IS added to blank plasma.

Intraday precision, interday precision, and accuracy were determined by analyzing QC samples at three concentrations (5, 50, and 1000 ng·mL^−1^, *n* = 6) on three separated days. The precision was defined as the relative standard deviation (RSD) of QC sample concentrations determined at 6 replicates, whereas accuracy was assessed as the percentage to the nominal concentration (%).

Recovery was determined by comparing the peak areas of the QC samples in the nonextracted control samples with those of the corresponding extracted samples. Three different concentration levels of each analyte (5, 50, and 1000 ng·mL^−1^, *n* = 6) were evaluated by analyzing six samples at each level. The same evaluation was performed for IS at 100 ng·mL^−1^.

The matrix effect was evaluated by comparing the peak areas of the postextracted blank plasma spiked mixed with working solutions to those of corresponding standard solutions at three QC levels, in triplicate; the same procedure was performed for the IS (100 ng·mL^−1^). The QC samples were subjected to short-term room temperature (2 h, 18°C), followed by a long-term freeze (30d, −20°C) and three freeze/thaw (−20°C–18°C) stability tests.

### 2.7. Pharmacokinetic Studies

Twelve healthy beagle dogs (male; weight: 8.5–12.5 kg) were used for the pharmacokinetic study to investigate the plasma concentration levels of apremilast. Food was withdrawn 12 h prior to experimentation. The beagle dogs received daily apremilast tablets by oral administration at a dose of 30 mg·day^−1^. Blood samples (approximately 3.0 ml) were collected from beagle dogs into heparinized tubes before dosing and at 0.17, 0.33, 0.50, 0.75, 1.00, 1.50, 2.00, 3.00, 4.00, 6.00, 9.00, 12.00, 24.00, 36.00, 48.00, 60.00, and 72.00 h postdosing, respectively. The plasma samples were obtained after immediately centrifugation at 4000 rpm for 10 min and then transferred into Eppendorf tubes before storage at −20°C until analyses. Animal experiment was conducted in accordance with the Guideline for Animal Experimentation, and the protocol was approved by the Institutional Animal Care and Use Committee (IACUC) of this institution (License number: K2015014).

Pharmacokinetic parameters were calculated by the statistical software “Drug and Statistics2.1.1” (DAS 2.1.1, Mathematical Pharmacology Professional Committee of China) and “Statistical Product and Service Solutions16.0” (SPSS 16.0, SPSS Inc, Chicago, IL, USA). The paired Student's *t*-test (*C*_max_, AUC, and CLz) and the Wilcoxon test (*t*_max_) were used for statistical analysis. Three dose comparisons conducted by one-way ANOVA followed by Turkey's multiple comparisons test differences associated with *p* values less than 0.05 were considered to be statistically significant.

## 3. Results and Discussion

### 3.1. Liquid Chromatography and Mass Spectrometry Conditions

**Figure 2:**
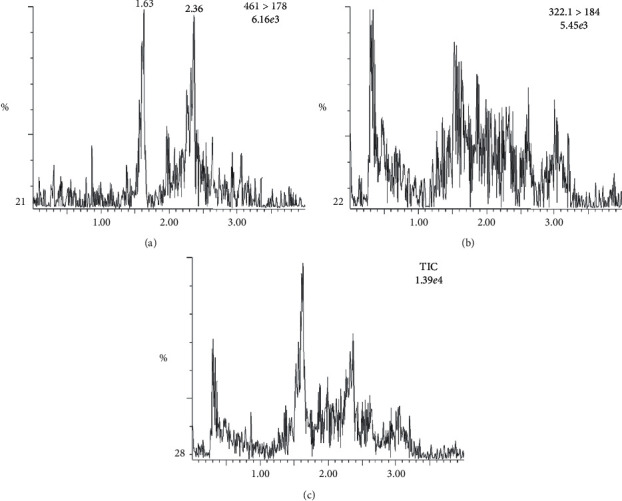
Representative chromatograms of blank plasma sample from a beagle dog.

Method development starts with the optimization of chromatographic conditions including column type and mobile phase composition. Since smaller diameter columns were commonly used for UPLC-MS-MS, three types of Waters ACQUITY UPLC at BEH columns with 1.7 *μ*m diameter were employed and compared in our investigations. BEH HILIC column was first excluded due to the unsatisfactory peak shape of apremilast. Besides, the Rt of apremilast on the BEH Shield RP18 column (1.49 min) was more appropriate than that of the BEH C18 (<1.00 min) regardless of their similar selectivity and the peak shape achieved for apremilast. Thus, to a consideration of adequate separation of the analyte to aqueous phase interfering substances (Rt < 1 min), a Waters ACQUITY UPLC at BEH Shield RP18 column was applied in the current analytical method with the total run time of 4.0 min. In addition, apremilast demonstrated a better response when the aqueous phase contained 5 mM (final concentration or not) ammonium formate instead of 5 mM ammonium acetate in combination with 0.1% (v/v) formic acid. Moreover, there was no obvious difference on the sensitivity between 5 mM ammonium formate solution and 10 mM ammonium formate solution, and 5 mM ammonium formate solution was used to reduce wastage of the volume. To sum up, liquid phases consisted of water and methanol containing 5 mM ammonium formate were employed in our current study. With the chromatographic conditions described above, apremilast and IS were eluted at the retention times of 1.49 min and 1.70 min, respectively (Figure 2).

In the early stage of the method development, standard solutions of the apremilast and IS at the concentrations of 1 *μ*g·mL^−1^ were injected into the mass spectrometer, respectively. It was noticed that the signal intensities of both apremilast and IS were enhancers in the positive mode in comparison to the negative mode, which might be attributed to the better electrospray ionization of positively charged apremilast under the positive mode. Therefore, the ESI in the positive ion mode was employed for the quantification of apremilast in the current study. The daughter scan mass spectrum of [*M* + *H*]^+^ showed the detail information of characteristic productions at m/*z* 461.3 and 322.1 for apremilast and clopidogrel, respectively. As shown in Figure 1, the most sensitive and stable product ions were m/*z* 178.0 and 257.2 for apremilast and m/*z* 184.0 for IS, respectively. The ESI parameters including capillary voltage, cone voltage, collision energy, gas flow-rate, and source temperature were optimized to maximize the MS response. The flow-rate of gas (desolvation gas, cone gas, and collision gas) and source temperature had minimal effects on the MS response.

### 3.2. Validation Procedures

#### 3.2.1. Specificity

**Figure 3:**
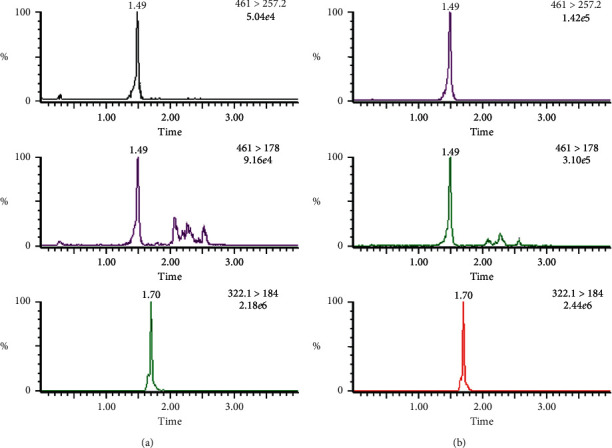
Representative chromatograms of apremilast and its IS in (A) blank beagle dog plasma spiked with working solutions at 2 ng mL^−1^ of apremilast and 100 ng L^−1^ of IS and (B) beagle dog plasma samples collected at 0.5 h after oral administration of apremilast at 30 mg·day^−1^.

In the present study, the specificity and selectivity have been studied by using independent plasma samples from six different dogs. Figure 2 shows representative chromatograms of blank (uncontaminated) extracts, and Figure 3 shows blank sample spiked with apremilast standard at LLOQ and real plasma sample with analytes. There is no significant endogenous interference from plasma found at Rt of analyte or the IS.

Autosampler carryover was determined by injecting the highest calibration standard and then a blank sample. No peaks were observed at the retention times of the analytes and IS on the chromatogram of blank plasma after subsequent injection of the highest calibration standard.

#### 3.2.2. Linearity and LLOQ

The calibration curve which concentration ranged from 2 to 3000 ng·mL^−1^ was linear in beagle dog plasma. The calibration curve for apremilast generated during the validation was *y* = 0.0126*x* + 0.0072 (*r* = 0.9979), where *y* represents the peak area ratio of apremilast to IS, and *x* is the nominal concentration of the analyte. The LLOQ of apremilast was 1 ng·mL^−1^ with acceptable precision (RSD) of 5.5% and accuracy (RE) of 1.9%.

#### 3.2.3. Precision and Accuracy

The intraday and interday precision and accuracy of the assay are summarized in Table 1. The intraday and interday precisions ranged from 3.3% to 5.9% and 4.6% to 8.3%, respectively; and intraday and interday accuracy was between 92.4% and 100.1% for apremilast.

#### 3.2.4. Recovery and the Matrix Effect

The extraction recoveries of apremilast at 5, 50, and 1000 ng·mL^−1^ ranged from 87.4% to 97.4% (Table 2). The absolute matrix effects were between 105.9% and 108.1%, indicating the matrix effect could be negligible under the experimental conditions.

#### 3.2.5. Stability Studies

The stability results indicated that apremilast was stable at room temperature up to 2 h with less than 15% deviation from initial concentrations. And the analyte was also stable after three freeze-thaw cycles and storage at −40°C for 3 months with less than 10% deviation from the initial concentrations. Those results were well within the acceptable limit, indicating that the analyte determined was sufficiently stable during the analysis.

### 3.3. Application to Pharmacokinetic Study in Beagle Dogs

**Figure 4:**
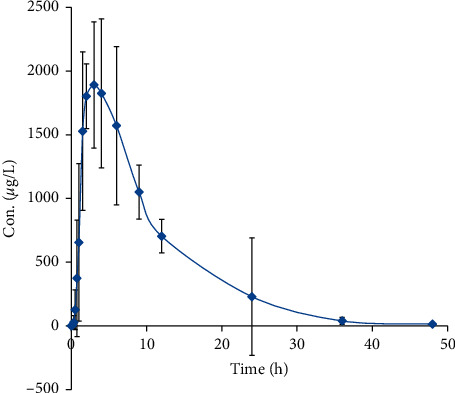
Mean plasma concentration versus time profile of apremilast in beagle dog after its oral administration at 30 mg·day^−1^ *y* (*n* = 12).

The analytical method described above was applied to the pharmacokinetic study of apremilast after its tablets oral administration at 30 mg kg^−1^ (day) in twelve male beagle dogs. It was noticed from the mean plasma concentration versus time profile of apremilast shown in Figure 4 that apremilast was well absorbed with the maximum plasma concentration at around 3.04 h. Afterwards, the apremilast was rapidly distributed to the peripheral tissues, leading to low plasma concentrations which can be barely detected after 48 h. The pharmacokinetic parameters including *t*_1/2_, *t*_max_, *C*_max_, AUC_last_, AUC_INF_, *V*_z_/F (apparent volume of distribution), and *C*_l_/F (clearance) were calculated by DAS and are listed in Table 3. The ratios of *C*_max_ and AUC in male beagle dogs for apremilast are 0.09.

## 4. Conclusions

In this study, we have validated a UPLC-MS-MS method for the quantification of apremilast after liquid-liquid extraction from beagle dog plasma. Validation results show that there is no significant matrix effect on analyte and selected IS. The reported method was successfully applied to the pharmacokinetic study of apremilast after its oral administration in beagle dogs. The mean t1/2Z was 5.41 h for 30 mg·day^−1^ for beagle dogs after oral administration. The AUC0-t increased linearly from 3.51 to 1802.13 *μ*g L^−1^*∗*h after administration of a single doses.

## Figures and Tables

**Table 1:** Precision and accuracy of apremilast in beagle dog plasma.

Nominal concentration (ng mL^−1^)	Intraday determination (*n* = 6)			Interday determination (*n* = 6)		
	Determined concentration (ng mL^−1^)	Accuracy (%)	R.S.D. (%)	Determined concentration (ng/mL)	Accuracy (%)	R.S.D. (%)
5	5.00	100.1	5.9	4.64	92.8	5.5
500	461.97	92.4	5.3	475.99	95.2	8.3
1000	935.94	93.6	3.3	900.56	90.1	4.6

**Table 2:** Extraction recoveries of apremilast and clopidogrel in beagle dog plasma.

Compound	Concentration (ng/mL)			Recoveries (%, *n* = 6)	RSD (%)
	Nominal	Determined/Before extraction	Determined/After extraction		
Apremilast	5.00	4.81	5.51	87.4	5.9
	50.00	46.05	49.08	93.8	5.3
	1000.00	842.08	864.48	97.4	3.3

**Table 3:** Pharmacokinetic parameters of apremilast after its single oral administration at 30 mg day^−1^ in beagle dogs (mean ± SD, *n* = 5).

Pharmacokinetics parameters	Apremilast
AUC_(0-t)_ (*μ*g L^−1^*∗*h)	22650.98±
AUC_(0-∞)_ (*μ*g L^−1^*∗*h)	22758.56±
*t*_1/2z_ (h)	5.41±
*T*_max_ (h)	3.04±
*V*_z/F_ (L)	11.28±
CL_z/_F (L h^−1^)	1.52±
*C*_max_ (ug L^−1^)	2148.33±

Blood samples (3.0 ml) were collected in heparinized tubes before dosing and at 0.17, 0.33, 0.50, 0.75, 1.00, 1.50, 2.00, 3.00, 4.00, 6.00, 9.00, 12.00, 24.00, 36.00, 48.00, 60.00, and 72.00 h postdosing, respectively. Plasma samples were obtained after immediate centrifugation of collected blood at 4000 rpm for 10 min.

## Data Availability

The raw/processed data required to reproduce these findings cannot be shared at this time as the data also form part of an ongoing study.

## Abbreviations

UPLC-MS-MS: Ultra-performance liquid chromatography-tandem mass spectrometry
MRM: Multiple reaction monitoring mode
PsA: Psoriatic arthritis
Th1: T helper 1 cells
Th17: T helper 17
PALACE: Psoriatic arthritis long-term assessment of clinical efficacy
IS: Internal standard
Rt: Retention times.
